# Seasonality, Moisture, and Host Community Structure of Haemaphysalis Ticks in a Subtropical Urban Mosaic in Hong Kong, China

**DOI:** 10.1002/ece3.73140

**Published:** 2026-02-23

**Authors:** Mathew Seymour, Kwan Wong

**Affiliations:** ^1^ The University of Hong Kong School of Biological Sciences Hong Kong China

**Keywords:** barcoding, *Haemaphysalis formosensis*, *Haemaphysalis hystricis*, host preference, iDNA, urban ecology

## Abstract

Ticks (Ixodida) are ecologically and epidemiologically important parasites, yet their diversity, host associations, and environmental drivers remain poorly resolved in many parts of the world, including subtropical and urban Hong Kong. Here, we combined wet‐season spatial surveys (23 sites in 2023) with monthly temporal sampling (four sites across 11 months in 2024) to characterize the spatiotemporal dynamics of ticks in Hong Kong. Ticks were collected using standardized drags, CO^2^ traps, and opportunistic sampling; adults were identified morphologically and validated via COI barcoding. Vertebrate host use was inferred through iDNA metabarcoding of tick abdomens. Our findings provide the first documented widespread occurrence of *Haemaphysalis hystricis* and *Haemaphysalis formosensis* in Hong Kong. Additionally, we provide initial insights into local life stage‐specific seasonality dynamics, whereby adult peaks in late winter–spring, nymphs are elevated in the cool dry months, and larvae noticeably surge during the wet season. In assessing the potential environmental drivers, adult abundance was most strongly associated with moisture (relative humidity and dew point). Presence‐only models suggested additional contributions from porcupine (*Hystrix* spp.) occurrence and temperature. iDNA analysis suggests primarily mammalian host feeding (73.7%), specficially wild boar (*Sus* spp.) and porcupines being the most frequent (43.4%), with additional detections of civets, dogs, cattle, and several bird families. In general, *H. hystricis* exhibited a broader host spectrum than *H. formosensis* . Overall, these results indicate that moisture availability and mammal host communities influence *Haemaphysalis* tick distributions across Hong Kong's mosaic landscape. Future consideration should be made for expanding spatial and temporal surveillance, integrating microhabitat moisture and host density data, and coupling ecological surveys with pathogen screening to inform One Health surveillance and management.

## Graphical abstract

We integrated field surveys, DNA barcoding and integrative taxonomy, iDNA, and ecological modeling to resolve the distributions, seasonality, and host use of *Haemaphysalis* ticks across Hong Kong. Moisture was indicative of adult activity, and iDNA suggested predominant feeding on wild mammals, particularly wild boar and porcupine. The One Health insights from this initial surveillance provide key preliminary guidance and risk communication at human–wildlife interfaces.

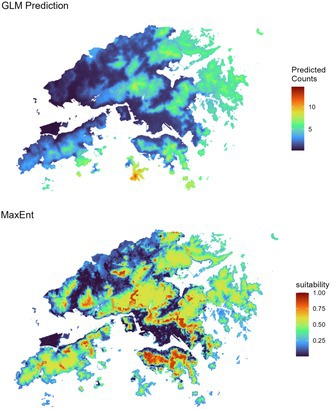

Understanding the ecological dynamics of parasites is fundamental to unraveling the complex relationships that shape biodiversity and ecosystem functioning. Parasites, including ticks (order Ixodida), play pivotal roles in regulating host populations, altering community structure, and mediating energy flow within ecosystems (Hudson et al. 2002; Poulin 2014). Despite their ecological importance, the spatial and temporal distributions of parasites remain poorly characterized, particularly with regards to changing environments (e.g., climate or landuse change) (Estrada‐Peña et al. 2014). Investigating the natural drivers of parasite occurrence, including habitat heterogeneity, host availability, and climatic variables, not only enhances our understanding of parasite ecology but also provides predictions of disease emergence and transmission (Ogden and Lindsay 2016; Randolph 2009). Insights into parasite‐linked pathogen projections are especially relevant within the One Health framework, which emphasizes the interconnectedness of human, animal, and environmental health (Destoumieux‐Garzón et al. 2018). Elucidating the factors that shape parasite diversity and distribution is therefore critical for anticipating and mitigating risks to wildlife conservation, livestock production, and public health in an era of rapid environmental change.

Spatial and temporal dynamics are fundamental aspects of parasite ecology, shaping patterns of infestation, transmission, and ultimately disease risk across landscapes and seasons. Ticks are hematophagous parasites with a broad spectrum of vertebrate hosts, including livestock, wildlife, and humans. They are recognized as major vectors of pathogens affecting both human and animal health, being second only to mosquitoes in their global epidemiological impact (Jongejan and Uilenberg 2004; Zhao et al. 2021). In general, tick abundance and infection prevalence vary drastically with changes in habitat structure, including vegetation composition, and environmental factors impacting tick survivability across their different life stages (Allan et al. 2003; Estrada‐Peña et al. 2012). Populations of ticks are often concentrated near semi‐urban and natural interfaces where humidity is favorable and preferable hosts are abundant (Boulanger et al. 2019; Marshall et al. 2025). Seasonal population and reproductive cycles are equally pronounced, with direct influences being attributed to changes in temperature and moisture, typically with adults being more prevalent in warmer and more humid time periods (Boulanger et al. 2019). Subsequently, inter‐annual variability in precipitation and humidity can alter tick ecology year‐to‐year, requiring risk maps to be updated routinely (Estrada‐Peña et al. 2014). These intertwined spatial and temporal processes generate shifting mosaics of tick‐borne disease hazards, underscoring the need for routine high‐resolution monitoring and predictive models that assimilate landscape metrics, climate trajectories, and host community dynamics (Ostfeld and Brunner 2015).

In the subtropical climate of Southeast Asia, tick biodiversity is largely understudied, but known to be dynamic and shaped by the diverse habitats, complex biogeography, and past and ongoing rapid habitat and environmental change (Zhang, Zheng, et al. 2019). Advances in surveillance and molecular diagnostics have led to the discovery of many new tick species in the region, including a growing array of tick‐associated pathogens (Zhao et al. 2021). Recent research shows that many species of ticks in China are currently expanding, including species with known disease vectors, such as *Haemaphysalis longicornis* and *Rhipicephalus microplus* , driven by increasing climate variability, land use change, and changes in distribution and movement patterns of wildlife and domestic hosts (Zhang, Zheng, et al. 2019). In East and Southeast Asia, *Haemaphysalis* species are particularly widespread, including *H. longicornis* , *H. hystricis* , and *H. formosensis* , with pronounced life stages showing seasonal dynamics. *Haemaphysalis* nymphs generally peak after the warmer months (fall and late wet season) with larvae peaking in spring and early wet season, followed by adults peaking in the warmer summer and wet season months (Meng et al. 2016; Zhang, Zhang, and Liu 2019; Zhao et al. 2021). Host preference is fairly broad for *Haemaphysalis*, including known parasitization of mammals, birds, and occasionally humans (Heath 2016). *Haemaphysalis* are also recognized as competent vectors of several zoonotic pathogens, including Rickettsia, Babesia, and various viruses (Wu et al. 2024; Zhang, Zhang, and Liu 2019). Studies from southern China and Japan have also linked *Haemaphysalis* activity to saturation deficit and changes in vegetation structure (Estrada‐Peña et al. 2012; Gilbert 2021), but comparable data for subtropical, highly urbanized mosaics remain scarce.

Although ticks are recognized in Hong Kong and have been documented by the Food and Environmental Hygiene Department (FEHD) since 1999, there is little comprehensive understanding of their distribution, diversity, or ecology. Currently there has been no document attempt to survey tick prevalence across Hong Kong, with the current knowledge largely restricted to veterinary or livestock records of infected animals. Among the ticks of potential importance, the genus *Haemaphysalis* stands out because of its ecological versatility and public health significance. Species such as *Haemaphysalis longicornis* and *Haemaphysalis hystricis* are widely distributed across East and Southeast Asia, including regions neighboring Hong Kong (e.g., Guandong), and are known for their adaptability to various habitats occurring within Hong Kong, including rural grasslands, forests, and peri‐urban green spaces (Zhao et al. 2021, 2020). Despite their likely presence and potential importance in Hong Kong, there remains a lack of systematic ecological surveys to confirm the residency, distribution, and host associations of *Haemaphysalis* ticks within the territory. The limited existing evidence of ticks in Hong Kong has instead focused on other genera, including *Ixodes* and *Rhipicephalus*, with research largely focused on potential disease vectors linked to pet and domesticated animal health (Hussain et al. 2023). Given the demonstrated adaptability and vector competence of *Haemaphysalis* in neighboring regions, detailed field surveys and molecular identification efforts are urgently needed to elucidate their presence, ecology, and potential public health implications in Hong Kong.

Invertebrate‐derived DNA (iDNA) is an innovative approach in ecological and epidemiological research, enabling the identification of vertebrate hosts from the blood meals of hematophagous invertebrates such as ticks, mosquitoes, and leeches. By extracting and sequencing trace amounts of vertebrate DNA contained within these blood meals, researchers can non‐invasively determine the recent feeding history of individual invertebrates and, by extension, assess patterns of host use and preference across time and space (Rodríguez‐Castro et al. 2023; Schnell et al. 2012). This approach offers a powerful complement to traditional methods of host identification, which often rely on direct observation or labor‐intensive trapping and examination of hosts (Alcaide et al. 2009). iDNA has proven particularly valuable for understanding the ecological dynamics of vectors like ticks, providing insights into which wildlife or domestic animals are most frequently parasitized and thus may play critical roles in the maintenance and transmission of tick‐borne pathogens (Massey et al. 2022). Through iDNA analysis, researchers can build a more comprehensive picture of host–parasite networks, assess changes in host availability or preference, and better predict the risk of zoonotic disease emergence (Massey et al. 2022). iDNA metabarcoding has been used to identify vertebrate hosts of ticks in Europe and the Americas, revealing host‐specific patterns of pathogen risk (Alkathiri et al. 2024; Carrera‐Faja et al. 2025). However, no comparable iDNA‐based host characterization exists for *Haemaphysalis* ticks in southern China or Hong Kong, leaving a major gap in our understanding of how local wildlife (e.g., wild boar, porcupines) support tick populations.

This study aimed to advance the understanding of tick ecology in Hong Kong by pursuing three key objectives. First, we determined the biodiversity of ticks across Hong Kong, characterizing both the spatial and temporal dynamics of tick populations in this sub‐tropical region. We hypothesized that (H1) tick species richness is currently underestimated and positively associated with habitat complexity and lower anthropogenic disturbance, reflecting higher vertebrate host diversity and more stable microclimates. Second, we investigated whether environmental patterns, specifically temperature and humidity, influenced the occurrence and distribution of tick species throughout Hong Kong. We predicted that (H2a) tick presence and community composition are strongly associated with warm, humid conditions and low saturation deficit, and (H2b) that these relationships are modulated by vegetation structure and urbanization, which alter microclimate and host access at fine spatial scales. Third, we evaluated the potential host range of natural tick populations using invertebrate‐derived DNA (iDNA) analysis of tick blood meals. We hypothesized that (H3) tick genera found in Hong Kong parasitize a broad assemblage of vertebrates, with host use biased toward locally abundant medium‐sized mammals (e.g., wild boar, porcupines, free‐roaming dogs).

## 1 Methods

### 1.1 Field Surveys

**FIGURE 1:**
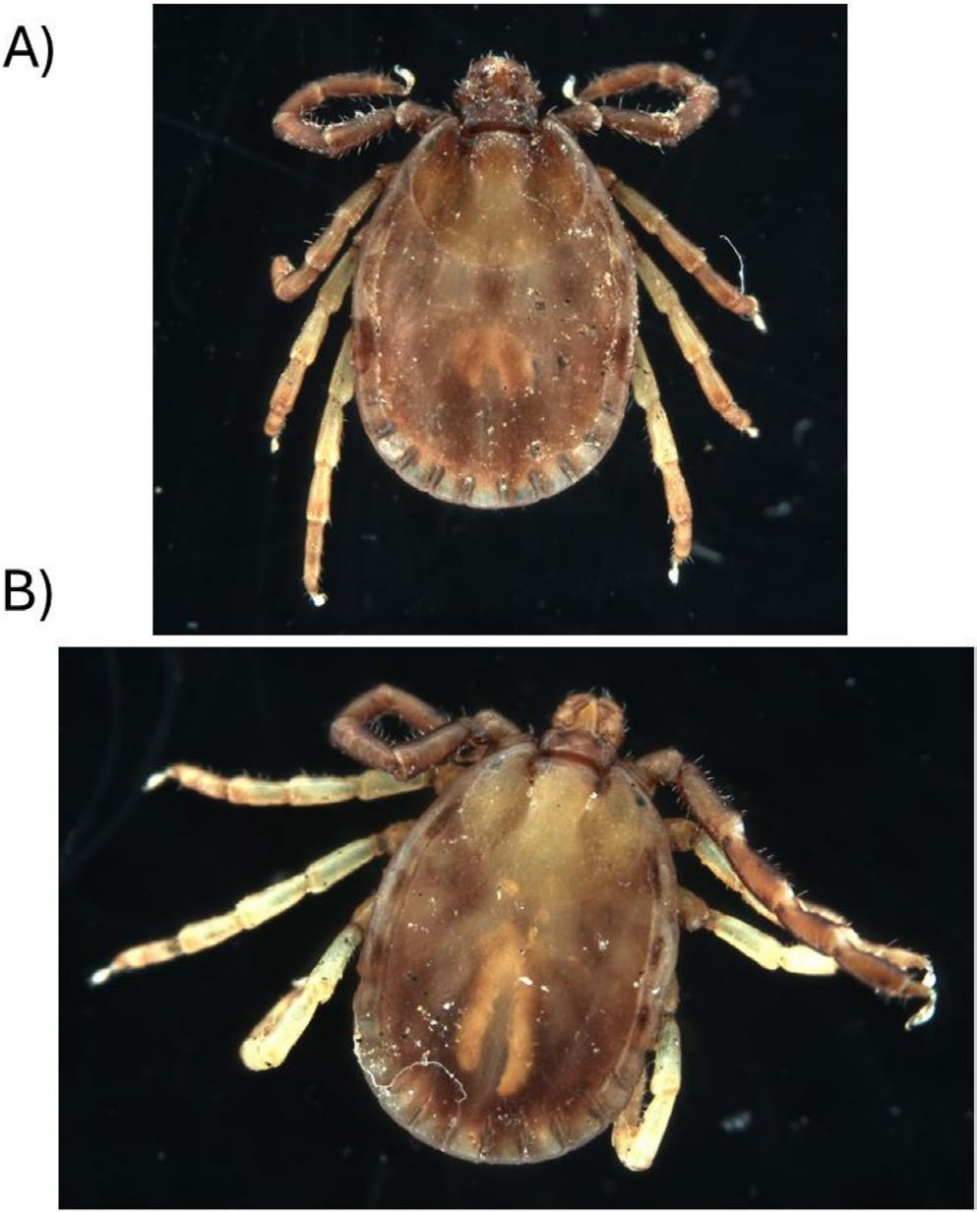
*Haemaphysalis hystricis* (A) and *Haemaphysalis formosensis* (B).

**FIGURE 2:**
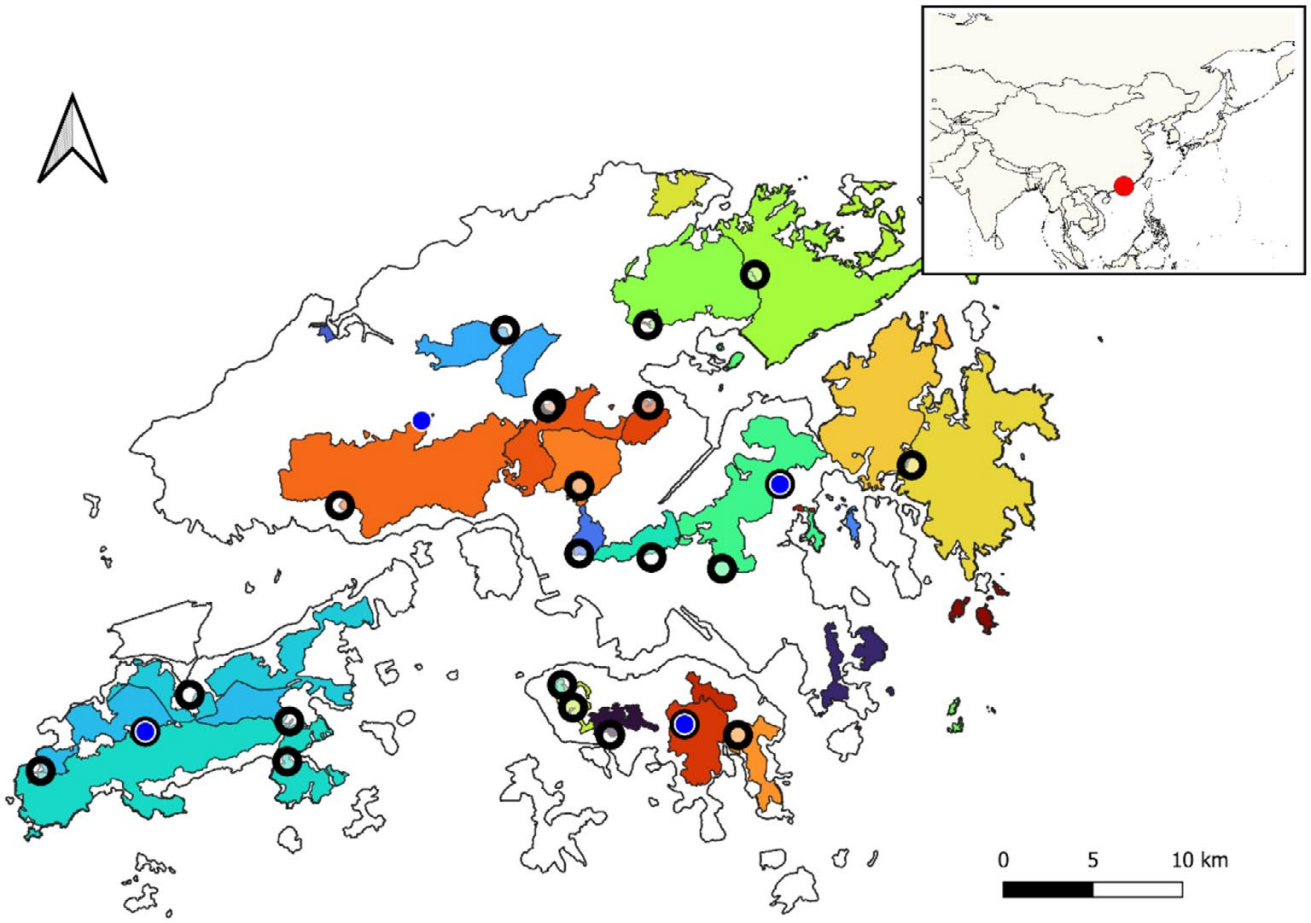
Map depicting Hong Kong with the 2023 (circles with black outline) and 2024 (blue points) sampling sites. The colored regions show the country park coverage (e.g., non‐urbanized area) within Hong Kong, with each of the colored regions showing a unique administrative boundary. The insert shows the location of Hong Kong within the larger region of Southeast Asia.

Hong Kong ticks (Figure 1) were surveyed in 2023 and 2024. For 2023, we surveyed 23 sites during the wet season from June to October. For 2024, we surveyed 4 sites monthly for 11 months from January 2024 to December 2024. Sites for the 2023 survey were selected across country parks (i.e., non‐urban areas), with the intended effort to maximize the spatial extent across Hong Kong within logistic constraints (Figure 2). The 2024 survey sites were selected across the extent of the 2023 survey effort where ticks were successfully collected from in 2023. We note that this is a non‐uniform sampling design, which should be taken into consideration when interpreting the results from this initial study. For both surveys, the same sampling methods were employed as follows. Survey sites included three subsites that were separated by approximately 100 m within each subsite, we performed tick drags consisting of 30–60 min using a 1 × 1‐m white cloth drag through the underbrush. Ticks, including adults, nymphs, and larvae, were collected off each tick‐drag and preserved in 90% Ethanol for later identification and molecular analysis. At each subsite, a passive CO_2_ trap was also placed, consisting of a thermos placed on its side and filled with CO_2_, left open to the air. The thermos was placed in the center of a 1 × 0.5 m cloth with the outer perimeter covered in double‐sided tape. The trap was left overnight, and ticks were collected off the double‐sided tape the following morning. Ticks were also opportunistically collected during the sampling if they were observed on vegetation.

Environmental data, including temperature, dew point, and humidity, were obtained from the Hong Kong weather observatory, which provides daily climate data from approximately 60 weather stations. For each site, we selected the nearest weather station to determine the environmental conditions for the day the sampling took place.

After collection, ticks were sorted into adults, nymphs, and larvae, and their abundances were recorded per site and sampling method. Adults were morphologically identified to species using taxonomic keys (Ernieenor et al. 2017; Intirach et al. 2023) and consultation with morphological experts. We further verified the morphological identification via COI barcoding, whereby 48 adults that were identified morphologically and photographed priorwere cut in half for DNA extraction using a Universal Genomic DNA Kit (Beijing Science & Beyond Technologies). COI barcoding was then carried out using the standard Folmer forward and reverse primers (Folmer et al. 1994; Seymour et al. 2024). Sequencing in both directions was conducted by Genewiz Ltd. (China) via Sanger sequencing on an ABI PRISM 3730xl DNA Analyzer. Subsequent sequence trace files were trimmed of low‐quality ends, and forward and reverse reads were assembled into contigs using Geneious 11.0.20.1 (Biomatters Ltd.). Sequence alignments were performed using MUSCLE alignment within Geneious 11.0.20.1.

Invertebrate DNA (iDNA) was extracted from 63 adults and 12 nymphs to assess likely host associations. For each iDNA sample (i.e., individual tick), we extracted DNA as above, but only from the abdomen of each tick to increase the target DNA concentration in the iDNA extraction. Metabarcoding libraries were then constructed using a widely used 12S vertebrate marker targeting key potential host species (Riaz et al. 2011). Libraries were constructed using a two‐step protocol (Bohmann et al. 2022; Si et al. 2025) with PCR1 amplifying the 12S mini‐barcode region and PCR2 attaching unique sample identifiers and Illumina adapter sequences. Meaning each tick abdomen DNA extract was labeled uniquely to allow demultiplexing of individual tick iDNA downstream. Libraries were cleaned, quantified, and normalized prior to sequencing at the Centre for Panoramic Services at The University of Hong Kong, using an Miseq (Illumina) with 2 × 300 bp chemistry. Sequences were then demultiplexed and bioinformatically processed using cutadapt (Martin 2011) to remove primer ends and vsearch (Rognes et al. 2016) to merge paired reads, quality filter, determine amplicon sequence variants (ASVs), and generate the final frequency table. ASVs were then assigned taxonomic identities using the CRABS pipeline (Jeunen et al. 2023) to generate a locally curated reference library from available sequences in GenBank.

## 2 Statistical Analysis

All analyses and visualizations were performed in R (version 4.4.2) (R Core Team 2024).

Spatial variation in adult tick abundance was modeled using generalized linear models (GLMs) with a Poisson error structure. Predictor variables included environmental factors (urbanicity, humidity, dew point, maximum temperature) and spatial host occurrence (presence/absence raster for key host species). Model selection was performed through stepwise AIC reduction, and multicollinearity was assessed with variance inflation factors (VIF) using the car package (Fox and Weisberg 2019). Model fit was evaluated by examining residual diagnostics and summary statistics. The final model was used to generate spatial predictions of adult tick abundance across Hong Kong, visualized as a prediction raster with sampling locations overlaid.

For the temporal survey data, we modeled the abundance of adult individuals (i.e., the response variable) using a generalized additive model (GAM) to assess the effects of temperature, month, and relative humidity. The model was fitted with a Poisson family and log‐link function using the mgcv package (Wood 2011). We used a forward model selection approach, with models evaluated using AIC. If a model was indicative of over‐fitting because of high explained variance or negative *R* ^2^, it was discarded. The final model included a smooth term for mean temperature (MeanTemp) and a tensor product interaction between month (Month) and mean relative humidity (MeanRelativeHumidity). The final model formula was:

$$\begin{array}{c} \text{Adult}\sim s\;\left(\text{MeanTemp},k=4\right)+\mathrm{te}\;(\text{Month},\;\text{MeanRelativeHumidity},\\ k=c(4,4)) \end{array}$$

To guard against overfitting the number of basis functions (*k*) was set to 4, thereby balancing flexibility and limiting the chance of overfitting risk (*n* = 42). Model performance was evaluated using the proportion of deviance explained and the unbiased risk estimator. We also inspected the k‐index tests and residual diagnostics using the function gam.check (Wood 2011). Models were rejected when any of the following occurred: (i) high explanatory power (> 80%) combined with negative *R* ^2^ or overdispersion ratio greater than 2, (ii) k‐index values less than 1, as an indication of saturation or (iii) observable patterns in residual‐fitted or QQ‐plots. Only models that met the above criteria were retained. Statistical significance for smooth terms was assessed using approximate *p*‐values.

### 2.1 iDNA Analysis

To minimize false positives from trace or spurious reads, we applied a proportional threshold, removing genus‐level detections representing less than 0.1% of total sample reads (Peabody et al. 2015). The resulting dataset was merged with sample metadata, including tick location, morphotype, and life stage. For each tick sample, we calculated the relative read abundance and log‐transformed abundance of each detected host genus. The top three host taxa per tick were reported on the basis of proportional read abundance, with secondary and tertiary host assignments masked if their proportional abundance was below 10%.

To assess whether vertebrate host community composition differed between the two tick species, we calculated pairwise Bray–Curtis dissimilarities between samples to quantify differences in vertebrate community composition. Permutational multivariate analysis of variance (PERMANOVA) was performed using the adonis2 function in the vegan R package, with tick species as the grouping factor. Statistical significance was assessed with 999 permutations.

### 2.2 Species Distribution Modeling

Remotely sensed raster layers for dew point, maximum temperature, and relative humidity were obtained for the wet season months (June–October). Rasters were stacked and averaged using the raster package to generate composite layers representing mean seasonal environmental conditions. An urbanicity raster (Gaussian‐smoothed index) was included to account for anthropogenic effects. Environmental values were extracted from these layers at each sampling site and appended to the tick survey dataset for subsequent modeling (Morgan and Guénard 2019).

To model potential spatial overlap between ticks and their vertebrate hosts, occurrence records for key host species identified via iDNA metabarcoding, *Hystrix brachyura* and *Sus scrofa* , were downloaded from the Global Biodiversity Information Facility (GBIF; www.gbif.org) using the rgbif package (Chamberlain et al. 2021). Records were filtered for spatial accuracy within the Hong Kong extent, transformed to WGS84, and rasterized to match environmental layers. Presence/absence raster for each host species was produced for use as predictors in spatial models.

Generalized linear models (GLMs) with Poisson error structure were fitted using urbanization, humidity, dew point, *H. brachyura* and *S. scrofa* as predictors. Multicollinearity among variables was evaluated using variance inflation factors (VIF) and pairwise correlations. Model selection was performed by sequentially removing predictors and comparing Akaike Information Criterion (AIC) values to identify the best‐supported model. The final model was used to generate spatial predictions of adult tick abundance across the study area. Predicted values were mapped as raster surfaces and visualized alongside observed sampling locations.

Maximum Entropy (MaxEnt) modeling was conducted using the dismo package (Hijmans et al. 2017). Tick presence locations were defined as sites with observed adult ticks; 10,000 background points were randomly sampled within the study area. Predictor variables included the averaged environmental rasters (dew point, humidity, maximum temperature), urbanicity, and host presence/absence rasters. All layers were resampled and stacked at a common spatial resolution. MaxEnt models were run with response curve and jackknife options enabled to assess variable importance. Model predictions were visualized as habitat suitability maps, with observed presence points overlaid for validation.

## 3 Results

**FIGURE 3:**
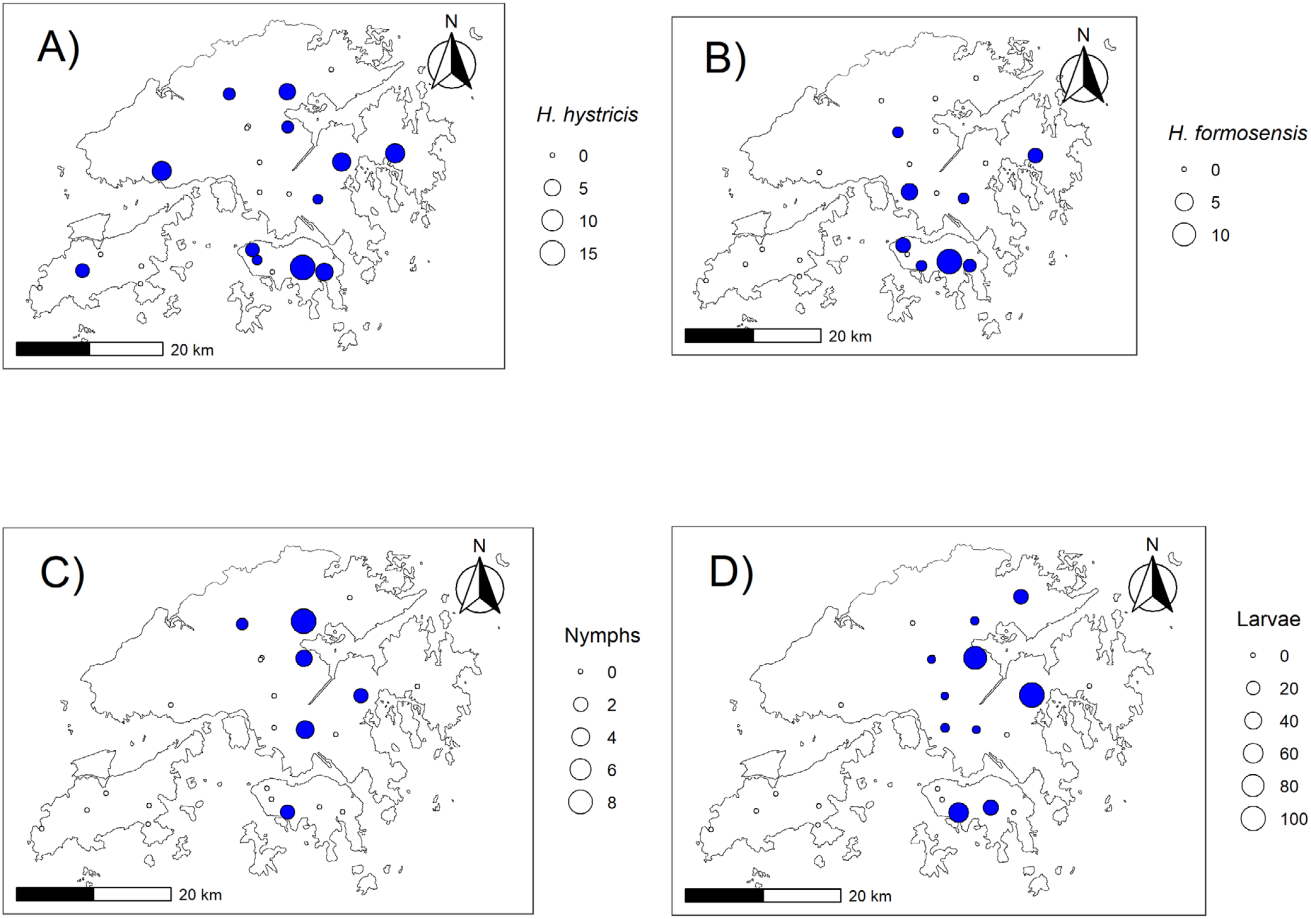
Spatial distribution of tick abundances across sampling sites in Hong Kong during the wet season. Each panel displays survey sites overlaid on a map of Hong Kong, with point size indicating the abundance recorded at each site and color denoting presence (blue) or absence (white) for each tick category. (A) Number of *Haemaphysalis hystricis* individuals per site. (B) Number of *Haemaphysalis formosensis* individuals per site. (C) Number of nymphs per site. (D) Number of tick larvae per site.

The 2023 field survey resulted in 435 ticks collected across the 23 sampling sites, including 88 adults, 21 nymphs, and 326 larvae. The adults were morphologically identified as *Haemaphysalis hystricis* and *Haemaphysalis formosensis* , which was further verified via COI barcoding. Of the 88 adults, 61 were identified as *H. hystricis* and 27 as *H. formosensis* . Spatial distribution of the ticks was generally widespread, with Lantau Island being notable as having few sampled ticks, with only *H. hystricis* 3 adults collected from 1 of the 5 Lantau sites and no nymphs or larvae collected from the 5 Lantau sampling sites. *H. hystricis* adults were the most widely observed, occurring across 12 sites, and *H. formosensis* occurred across 8 sites (Figure 3).

**FIGURE 4:**
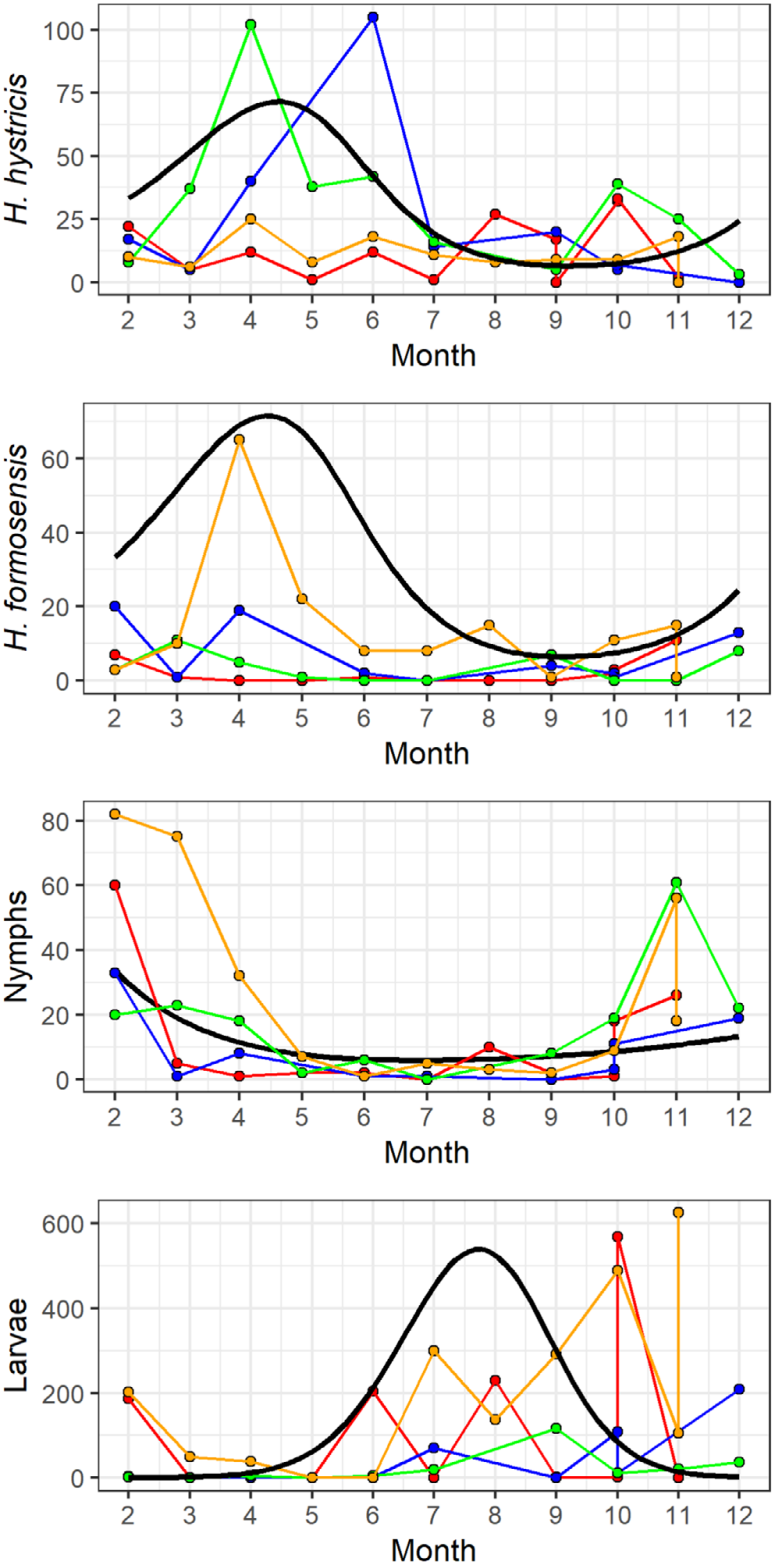
Seasonal dynamics of tick counts by life stage and site. Panels show the monthly counts of (A) *Haemaphysalis hystricis* , (B) *Haemaphysalis formosensis* , (C) nymphs, and (D) larvae from four study sites: Ma On Shan (red), She Shan Tsuen (blue), Tai Lam (green), and Tai Tam (orange). Points and colored lines represent observed values for each site; the solid black line in each panel shows the fitted values from the best‐fit Generalized Additive Model (GAM). Months are shown on the x‐axis (1 = January, 12 = December).

The 2024 temporal field survey resulted in 5824 ticks collected across the 4 sites and 11 monthly samples, including 4054 larvae, 673 nymphs, 816 *H. hystricis* adults and 281 *H. formosensis* adults. Temporal effects on tick abundances were noticeable and differed across life stages. Adults were observed throughout the year with noticeable increases during April and March for 3 of the 4 sites. Nymphs showed more pronounced temporal changes with greater abundances occurring in the dry season between November and February for all four sites. Larvae were less commonly observed from February to June and then increased rapidly from July to November, likely coinciding with the wet season (Figure 4).

For adults, the GAM explained approximately 45.3% of the deviance in adult abundance (adjusted *R* ^2^ ≈ 0.079, UBRE = 10.58, *n* = 42). Both mean temperature and the interaction between month and mean relative humidity had significant effects on adult abundance (Table 1). The smooth term for mean temperature had an estimated effective degrees of freedom (edf) of 2.99 and was highly significant (χ^2^ = 39.74, *p* < 0.001). The tensor product interaction between month and mean relative humidity was also highly significant, with an edf of 12.84 (χ^2^ = 296.87, *p* < 0.001).

**TABLE 1:** Summary statistics for the best‐fit generalized additive models (GAMs) describing the effects of environmental variables on tick counts by life stage. The models were fitted with a Poisson error distribution. Terms include smooth functions of mean temperature (s (MeanTemp)) and tensor product smooths of month and mean relative humidity (te (Month, MeanRelativeHumidity)). EDF = estimated degrees of freedom; Ref DF = reference degrees of freedom; Statistic = test statistic for the smooth term; *p*‐value = statistical significance of the term.

Term	EDF	Ref DF	Statistic	*p*
Adult				
s (MeanTemp)	2.99	3	39.7	< 0.01
te (Month, MeanRelativeHumidity)	12.8	13.8	297	< 0.01
Nymph				
s (MeanTemp)	2.98	3	48.7	< 0.01
te (Month, MeanRelativeHumidity)	14.3	14.5	258	< 0.01
Larvae				
s (MeanTemp)	8.94	9	877	< 0.01
te (Month, MeanRelativeHumidity)	15	15	2327	< 0.01

For nymph abundance, the GAM explained approximately 86.6% of the deviance (adjusted *R* ^2^ ≈ 0.89), with a UBRE of 2.905 (*n* = 42). Both mean temperature and the interaction between month and mean relative humidity had significant effects on nymph abundance (Table 1). The smooth term for mean temperature had an estimated effective degrees of freedom (edf) of 2.98 and was highly significant (χ^2^ = 48.7, *p* < 0.001). The tensor product interaction between month and mean relative humidity was also highly significant, with an edf of 14.26 (χ^2^ = 258.0, *p* < 0.001).

For larvae abundance, the GAM explained approximately 73.3% of the deviance (adjusted *R* ^2^ ≈ 0.725), with a UBRE of 53.258 (*n* = 42). Both mean temperature and the interaction between month and mean relative humidity had significant effects on larvae abundance (Table 1). The smooth term for mean temperature had an estimated effective degrees of freedom (edf) of 8.94 and was highly significant (χ^2^ = 877.1, *p* < 0.001). The tensor product interaction between month and mean relative humidity was also highly significant, with an edf of 15.00 (χ^2^ = 2327.1, *p* < 0.001).

Across all three life‐stage models, diagnostic checks in mgcv (gam. check) indicated no evidence of overfitting according to our a priori criteria: residual deviance/df was < 2, k‐index values were > 1, and residual and QQ plots showed no strong systematic patterns.

A Poisson regression analysis was conducted to identify the environmental and host‐related factors influencing adult tick abundance (Table 2). The most parsimonious model included humidity and dew point as significant predictors. The model estimates indicated that higher humidity (β = 0.302, SE = 0.097, *p* = 0.0018) and increased dew point (β = 0.653, SE = 0.317, *p* = 0.039) were associated with greater adult tick abundance. The intercept was significantly negative (β = −39.138, SE = 14.637, *p* = 0.0075), suggesting low baseline counts when predictors are at zero. Model comparison on the basis of AIC demonstrated that this reduced model provided the best fit among the tested models.

**TABLE 2:** Summary statistics for the generalized linear model (GLM) evaluating the effects of environmental variables on the spatial distribution of adult ticks across sampling sites. Shown are the model coefficient estimates, standard errors, test statistics, and *p*‐values for the two significant predictors retained in the final model: Relative humidity (“humid”) and dew point (“dew”). Both variables were positively associated with adult tick abundance.

Term	Estimate	Std error	Statistic	*p*
humid	0.30	0.97	3.12	< 0.01
dew	0.65	0.32	2.06	0.04

### 3.1 Host Preference

**FIGURE 5:**
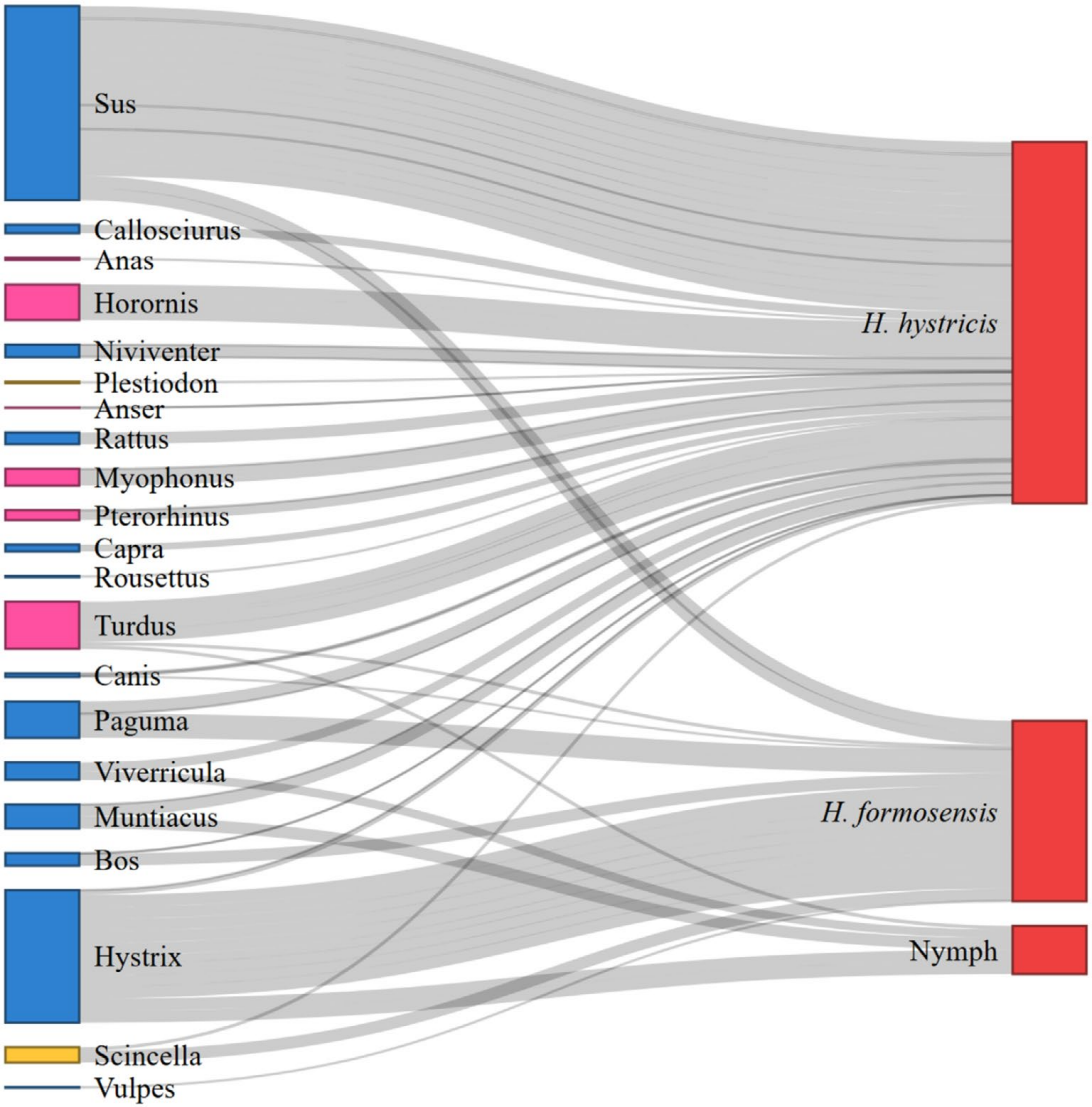
Sankey diagram illustrating the relative frequency of vertebrate host DNA detected in tick samples, grouped by tick morphotype. The left side of the diagram lists tick morphotypes ( *H. hystricis* , *H. formosensis* , and Nymph), whereas the right side shows vertebrate host genera detected in tick blood meals, colored by vertebrate class (Mammalia: Blue, Lepidosauria: Yellow, Amphibia: Green, Aves: Pink). The width of each flow is proportional to the relative abundance of host DNA sequences detected (proportion of total reads per tick).

PERMANOVA results indicated significant difference in host preference between the tick species (Table 3). *Haemaphysalis formosensis* (Figure 5) suggest a strong mammalian host preference (88.2%), with Porcupine (Hystricidae) the single most frequent taxon (50%), followed by Civets (Viverridae), Dog (Canidae), and Wild boar (Suidae) (each ~11%) and Cattle (Bovidae) (5.5%); birds and reptiles were rare (Turdidae 5.9%; Scincidae 5.9%). *Haemaphysalis hystricis* (Figure 5) suggest a broader host spectrum: 68.5% were mammals, 27.8% were birds, and 3.7% were reptiles. Suidae was the dominant host group (33.3%), with smaller contributions from the rodent group Muridae (7.4%), Canidae (5.6%), Viverridae (5.6%), and Bovidae (5.6%). Avian hosts included Turdidae (7.4%), Anatidae (5.7%), Muscicapidae (5.7%), Scotocercidae (5.7%), and Leiothrichidae (3.6%). Potential reptile hosts included Scincidae (3.7%). The five (i.e., very limited) nymph detections (Figure 5) were predominantly mammalian (80%) with one avian record (Turdus; 20%), with Hystricidae as the most common nymph host (40%). Overall, mammals were the principal host class (73.7%), followed by birds (22.4%) and reptiles (3.9%), and the two most commonly detected taxa overall were Suidae (26.3%) and Hystricidae (17.1%). Further assessment involved more specimens, targeted host field surveys, or iDNA with multiple additional markers to broaden the potential host pool are needed to evaluate these preliminary results.

**TABLE 3:** Results from a permutational multivariate analysis of variance (PERMANOVA) testing for differences in vertebrate host community composition detected in tick samples between species ( *H. hystricis* , *H. formosensis* ) and life stages. Degrees of freedom (DF), sum of squares, *R* ^2^, F‐statistic, and *p*‐value are shown for the model and residuals. A significant effect of tick species/life stage was detected (*p* < 0.01), indicating that host preference differs between groups.

	DF	Sum of squares	*R* ^2^	F	*p*
Model	2	1.89	0.09	2.16	< 0.01
Residual	46	20.08	0.91		
Total	48	21.96	1.00		

### 3.2 Species Distribution Models

**FIGURE 6:**
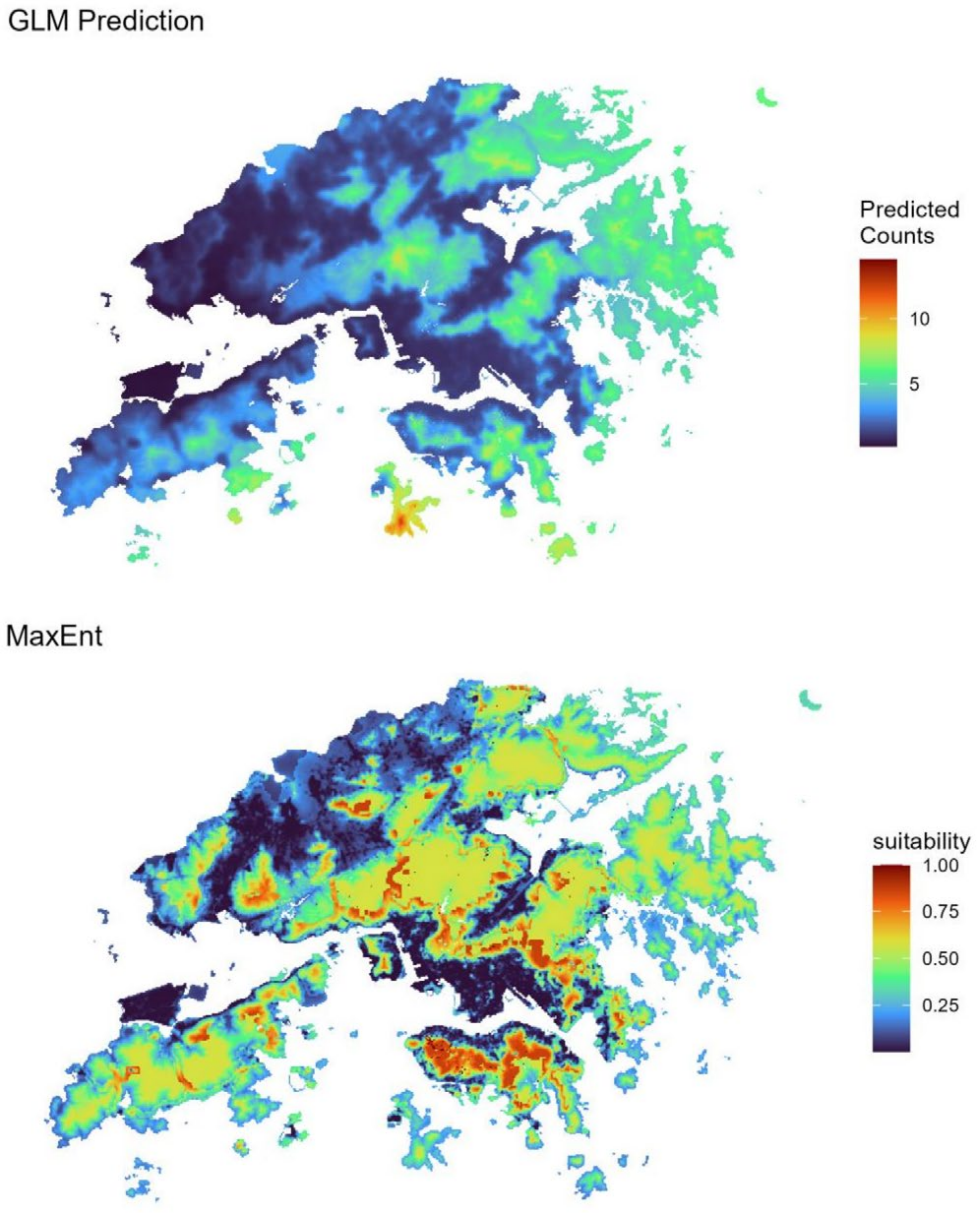
Spatial prediction of adult tick distributions in Hong Kong: Generalized linear model (GLM) versus MaxEnt. Top panel: Predicted abundance of adult ticks across Hong Kong, as estimated by a generalized linear model (GLM) fit to environmental predictors. Colors indicate predicted counts, with warmer colors representing higher predicted abundances. Bottom panel: Predicted habitat suitability for adult ticks generated using the MaxEnt species distribution modeling approach. The color gradient represents relative habitat suitability, with warmer colors indicating areas of higher predicted suitability. In both panels, predictions are mapped across the extent of Hong Kong.

The best GLM predicted species distribution model included dew point and relative humidity, with urbanization, *Hystrix brachyura* , and *Sus scrofa* being excluded during model fitting on the basis of AIC model selection. Spatial projections of the final GLM across the environmental layers produced a coherent prediction surface, with hotspots broadly coinciding with clusters of observed adult occurrences. The overlay of sampling points showed that most potential host presence sites fell within areas of moderate‐to‐high predicted tick abundance (Figure 6). Together, these results indicate that moisture‐related conditions and *Sus scrofa* presence are key correlates of adult tick abundance across the sampled sites in Hong Kong.

The MaxEnt model used 15 training presences and 9683 background points across six predictors (dew point, maximum temperature, relative humidity, urbanization, *Hystrix brachyura* , *Sus scrofa* ). Model performance was moderate (training AUC = 0.79; regularized training gain = 0.62). Variable contributions ranked *H. brachyura* as the strongest contributor (40.2%), followed by maximum temperature (32.5%) and urbanization (21.2%), with dew point (3.9%) and humidity (2.3%) contributing minimally, and *S. scrofa* being negligible (0.0%). Permutation importance highlighted dew point (45.8%) and *H. brachyura* (41.6%) as most influential, with humidity (12.6%) lower and temperature, urbanization, and *S. scrofa* near zero. Jackknife analyses were consistent, with *H. brachyura* removal generally reducing training gain the most (~ 0.45), whereas models restricted to *H. brachyura* or maximum temperature individually provided the highest gains (~0.25) relative to other predictors. Threshold diagnostics indicated that the 10th percentile training presence threshold (cloglog = 0.323) classified ~51.6% of the landscape as suitable with low training omission (6.7%), whereas the equal sensitivity–specificity threshold (cloglog = 0.479) reduced the suitable area to ~32.0% with higher omission (33.3%). The resulting suitability surface broadly encompassed observed adult occurrence points and identified additional warmer temperatures or *H. brachyura* ‐associated patches, providing a complementary, presence‐only perspective to the GLM abundance predictions. Both the GLM and MaxEnt predictive maps, however, are primarily exploratory, with further sampling and assessment needed to confirm the findings presented here.

The MaxEnt model demonstrated good predictive capacity, with a training AUC of 0.83, indicating strong discrimination between suitable and unsuitable habitats for adult ticks within the study region. The model's regularized training gain was 0.66, suggesting moderate model complexity and effective regularization to prevent overfitting. Variable contribution analysis revealed that the most influential environmental predictors were urban land cover (20.9%) and dew point temperature (11.4%), with urban contribution also exhibiting the highest permutation importance (49.9%). Other variables, such as dew point and maximum temperature, contributed substantially to the model, with dew point alone accounting for approximately 30% of permutation importance, underscoring its ecological significance. The Cloglog threshold value of 0.0793 was used to classify areas as suitable or unsuitable. The model predicted that approximately 80% of the study area falls within a suitable habitat on the basis of this threshold, with high suitability scores concentrated in urbanized and humid zones. This spatial pattern aligns with known tick ecology, where urban and humid environments favor tick proliferation.

## 4 Discussion

Our findings provide a first picture of *Haemaphysalis* tick ecology in Hong Kong, a subtropical, urbanized landscape, where moisture availability, host communities, and seasonal dynamics jointly structure tick abundance and occurrence. Across two complementary field efforts, an extensive wet‐season survey and a year‐long temporal study, adult *Haemaphysalis* ticks were found to be spatially heterogeneous and widespread, with a notable scarcity on Lantau Island, which should be further investigated to confirm. Life stages showed contrasting seasonal peaks consistent with known expectations. Additionally, this study novelly integrates site‐level tick life stages population data with species distribution modeling and iDNA metabarcoding. These analyses show that moisture‐related conditions are a primary environmental driver, whereas host associations with wild boar and porcupines likely mediate local hotspots and cross‐habitat connectivity in the region. Together, these results underscore the value of combining spatially explicit models with direct host‐use evidence to infer mechanisms behind tick distributions and to anticipate where and when human and animal exposure risks may be elevated.

The two *Haemaphysalis* species detected, *H. hystricis* and *H. formosensis* , are broadly distributed in East and Southeast Asia and well adapted to diverse habitats, including forest edges, shrublands, and peri‐urban green spaces (Ernieenor et al. 2017; Kwak and Ng 2022). In Hong Kong's mosaic of country parks, secondary forests, and urban interfaces, *Haemaphysalis* sp. tick ecology likely reflects a balance between microclimate refugia that reduce saturation deficits and host corridors that enable feeding and dispersal (Estrada‐Peña et al. 2012). *Haemaphysalis hystricis* is widely reported across southern China and neighboring regions, and is known for a relatively broad host spectrum and capacity to exploit ecotones; whereas *H. formosensis* , documented primarily in subtropical East Asia, is frequently associated with mammalian hosts and more specifically with forested habitats (Zhao et al. 2021). Both species have been implicated in the transmission of multiple zoonotic agents regionally, and their presence in Hong Kong is consistent with recent reports of expanding tick diversity and changing host communities across subtropical Asia (Wu et al. 2024; Zhao et al. 2021). The scarcity of adults on Lantau may reflect local differences in microclimate, vegetation, and host densities, which drive a unique island ecology (Dudgeon and Corlett 1994), and highlights the need to couple climatic predictors with localized habitat and host data to capture fine‐scale variation.

Temporal patterns in life stages were pronounced and support expected stage‐specific responses to climate and resource availability (Estrada‐Peña et al. 2012; Gilbert 2021). Adults were present year‐round, peaking during the early wet season, consistent with conditions of moderate temperature and sufficient humidity that favor questing and mating (Sonenshine and Roe 2014). Nymphs peaked during the dry season, indicating that cooler, drier months may align with developmental transitions from larvae or with seasonal host availability. The persistence in winter may imply nymphs use microhabitat refugia to buffer ambient saturation deficits (Perret et al. 2000). Larvae increased rapidly in the wet season, tracking higher humidity and vegetation growth that improve survival and host encounter rates (Estrada‐Peña et al. 2014). Temperature effects were significant across life stages, and the interaction between month and relative humidity captured the shifts in life‐stage development between larvae, nymphs, and adults (Sonenshine and Roe 2014).

Host‐use patterns derived from iDNA reveal species‐specific and stage‐specific associations that likely shape local tick distributions (Rodríguez‐Castro et al. 2023). *Haemaphysalis formosensis* was strongly mammal‐oriented, with porcupines dominating detections and wild boar, civets, dogs, and cattle contributing secondarily; avian and reptile detections were comparably rare (Zhang, Zhang, and Liu 2019). *Haemaphysalis hystricis* maintained a broader portfolio, including mammals, birds, and occasional reptiles, with wild boar prominent among mammals and multiple passerine and waterfowl families among birds (Zhang, Zhang, and Liu 2019). Nymphs were predominantly associated with mammals, with porcupine being a more frequent host, suggesting that juvenile stages may rely on mammal hosts even as adult *H. hystricis* exhibits more generalism (Sonenshine and Roe 2014). The host associations complement the spatial model results, whereby areas supporting wild boar and porcupines, and ecotones where birds and small mammals are abundant, may act as key habitats for tick life stage development and dispersal (Allan et al. 2003). It is crucial to note that read abundance in metabarcoding is not a direct proxy for feeding frequency, but the consistency of detections across taxa supports a genuine signal of host use relevant to the maintenance of tick populations and pathogen cycles (Deagle et al. 2019).

Moisture was found to principally influence adult *Haemaphysalis* sp. distributions, whereas host influences emerged more clearly in presence‐only modeling (Estrada‐Peña et al. 2012). Specifically, dew point and relative humidity were linked to aggregate hotspots that aligned with sites where adults were most prevalent, supporting expectations that saturation‐deficit constraints impact tick survival and questing (Perret et al. 2000). The MaxEnt model achieved moderate discrimination and, after accounting for collinearity, highlighted dew point and porcupine presence as potentially strong associations with tick spatial distributions. It is worth noting that temperature and urbanization did contribute during model fitting but showed lower permutation importance in the final solution, suggesting that additional research may be needed to further assess their direct impact. The analytical divergence between the GLM and MaxEnt models reflects the models' targets (abundance vs. occurrence), data structure (counts vs. small‐n presence‐only), and how each handles correlated predictors (AIC selection vs. regularization) (Zhou et al. 2025). Ecologically, the models suggest that moisture sets the broad envelope of suitability, whereas porcupine and wild boar presence help identify localized patches where adult ticks are more likely to occur or persist. Temperature may further modulate activity windows, and urbanization may act as a proxy for microhabitat edges rather than an independent driver (Allan et al. 2003; Zhao et al. 2021).

Although this study offers a much‐needed first assessment of tick ecology dynamics in Hong Kong, several caveats warrant caution and point to priorities for future work. Firstly, the presence‐only distribution modeling relied on 15 presences with a small fraction of missing predictor values. Future efforts can look to improve the accuracy and sensitivity of this initial assessment by utilizing a uniform sampling to reduce potential sampling bias. The findings presented here are primarily cursory, and caution should be given when considering the correlated predictors and the heuristic nature of the variable contributions, permutation importance when interpreting the results presented here. Additionally, the host rasters are derived from GBIF presence/absence data, which are a valuable resource but do not reflect actual population density, effort, or temporally concurrent movements. GBIF data are also very coarse resolution, likely underrepresenting the fine‐scale host heterogeneity that ticks experience, so care should be taken to infer directly from this initial assessment. Detection methods differ in sensitivity across life stages and microhabitats, and Lantau Island's apparent scarcity could reflect real ecological differences or sampling limitations. iDNA assignments depend on reference library completeness and can be subject to marker amplification biases. As such, future studies would benefit from a multi‐marker approach. Read proportions have been shown to be correlated with community relative abundances (Skelton et al. 2023), but may also potentially be influenced by variation in PCR amplification. Nevertheless, iDNA for assessing host specificity offers a reliable and cost‐effective means to evaluate likely hosts and is less environmentally invasive compared to host‐targeted trap surveys.

Our findings do have direct implications for stakeholders engaged in One Health surveillance in Hong Kong. The clear seasonal peaks of larvae in the wet season and adults in late winter–spring indicate predictable windows of heightened exposure risk for hikers, field workers, and domestic animals. Public health authorities (e.g., FEHD and Department of Health) can use this information to time public advisories, tick checks, and pathogen surveillance around these seasonal windows. Wildlife and country park managers can focus on monitoring and habitat management in areas where our models predict high moisture and co‐occurrence with wild boar and porcupines, particularly along popular trails and ecotones. Veterinarians and livestock managers can incorporate these spatial–temporal patterns into targeted tick control efforts. By coupling ecological insights with explicit recommendations on where and when to prioritize surveillance, our study provides a foundation for more efficient, evidence‐based tick‐borne disease management in Hong Kong.

Our integrative approach shows that moisture availability and mammalian host communities, particularly wild boar and porcupines, are central to the ecology of *Haemaphysalis* ticks in Hong Kong (Perret et al. 2000; Zhao et al. 2021). Stage‐specific seasonal peaks and species‐specific host portfolios create predictable windows and locations of elevated exposure risk (Sonenshine and Roe 2014) and the broad host use observed for *H. hystricis* is consistent with evidence that generalist taxa can stabilize community processes under oscillating environmental conditions, sustaining parasite–host interactions across heterogeneous landscapes (Seymour et al. 2020). These insights provide actionable guidance for targeted surveillance and management within a One Health framework (Destoumieux‐Garzón et al. 2018). Future work should expand spatial and temporal coverage, incorporate microhabitat moisture metrics and host density indices, and develop species‐ and stage‐specific models with cross‐validation to refine predictions. Coupling these ecological insights with pathogen screening will clarify the public health significance of Hong Kong's tick fauna and support targeted interventions to reduce tick‐borne disease risk in both wildlife and human communities.

## Author Contributions

**Mathew Seymour:** conceptualization (equal), data curation (equal), formal analysis (equal), funding acquisition (equal), investigation (equal), methodology (equal), project administration (equal), supervision (equal), validation (equal), visualization (equal), writing – original draft (equal), writing – review and editing (equal). **Kwan Wong:** data curation (equal), investigation (equal), methodology (equal), writing – review and editing (equal).

## Funding

Funding was provided by a Seed Fund for Basic Research grant to MS.

Contributions: Conceptualization: MS; field sampling: KW; laboratory and formal analysis: MS and KW; manuscript drafting and reviewing: MS and KW.

## Conflicts of Interest

The authors declare no conflicts of interest.

## Data Availability

Available at https://figshare.com/s/5bc12fe264b5e9d867bd.
